# Literacy Level and Executive Control in Healthy Older Peruvian Adults

**DOI:** 10.3389/fneur.2021.629048

**Published:** 2021-08-26

**Authors:** Marcio Soto-Añari, Norman López, Claudia Rivera-Fernández, Verónica Belón-Hercilla, Sara Fernández-Guinea

**Affiliations:** ^1^Laboratorio de Neurociencia, Departamento de Psicología, Universidad Católica San Pablo, Arequipa, Peru; ^2^Universidad de la Costa, Barranquilla, Colombia; ^3^Universidad Nacional de San Agustín de Arequipa, Arequipa, Peru; ^4^Departamento de Psicología Experimental, Facultad de Psicología, Universidad Complutense de Madrid, Madrid, Spain

**Keywords:** literacy level, executive control, aging, neuropsychology, dementia, education

## Abstract

**Introduction:** Early-life educational experiences are associated with cognitive performance in aging. Early literacy seems to improve executive control mechanisms, however, it is not clear whether early education would still be an advantage in countries like Peru, where access to and quality of education is highly variable.

**Aim:** Our objective was to analyze the association of literacy level with executive control factors.

**Method:** We evaluated 93 healthy older adults with a clinical protocol that included the Mini-Mental State Examination, the Geriatric Depression Scale and Global Dementia Staging. We also used a neuropsychological executive function battery which included the Trail-Making Test parts A and B, the Stroop Test, phonological and semantic verbal fluency tasks, Forward and Backward Digits, Numbers and Letters of the Wechsler Scale, and the Go/No-Go task. We used a principal component analysis for the dimensional reduction of the variables. To measure the level of literacy we used the word accentuation test (WAT).

**Results:** We observed statistically significant correlations between the principal components (PCs) of working memory, cognitive flexibility and inhibitory control with the WAT scores. Furthermore, we observed that processing speed and WAT predict the scores on PCs factors better than years of education and age.

**Conclusions:** Literacy level correlates more closely with better cognitive performance than years of education and thus, might improve executive control factors that could compensate and protect against brain changes in cognitive decline and dementia.

## Introduction

The rapid aging of the population is one of the most serious issues worldwide in this century (1). An estimated 2.1 billion people will be elderly by 2050. Eighty percentage will live in low- and middle-income countries, such as most Latin-American countries, and a high percentage will be illiterate, have difficulty in accessing education or have received poor quality education (1). This scenario will bring an increase in diseases and health disorders such as dementia (2), for which a low educational level is one of the main risk factors (3, 4), along with a low literacy level.

Early-life educational experiences have an important effect on cognition throughout development (5). The pioneering studies of Manly et al. (6, 7), demonstrated that literacy level was a more reliable predictor of cognitive decline in ethnic minorities and immigrants than years of education. Later studies confirmed these findings (8, 9). Therefore, the literacy level achieved, including not only the ability to read and write but also the competent use of information, seems to modulate the cognitive response in older adults with different ethnicities, through basic and complex cognitive mechanisms (10).

This modulation has been associated with basic attentional mechanisms such as processing speed, which is known as an important factor influencing cognition in aging (11) and executive control (12), which includes working memory, inhibitory control and cognitive flexibility (13). These mechanisms are activated in normal (14, 15) and pathological (16) cognitive aging to better cope with physiological changes or task demands (12, 17).

These executive control mechanisms are activated during a complex or novel task, or when processing resources are reduced, as in pathological aging (18). They activate brain processing networks that compensate for changes (19, 20). Other studies have suggested that these compensatory activations are not always positive, because it seems to have a threshold above which they could be rather inefficient, thus reflecting a greater level of deterioration (21).

Literacy level has been assessed through asking subjects to read low-frequent words, classically used for the estimation of premorbid IQ (22). It is assumed that the pronunciation of these words is associated with the literacy level achieved and that this in turn is correlated with IQ (23). For this, the Word Accentuation Test (WAT) has been used for Spanish speakers (24). This has shown itself to be useful in studies with neuropsychiatric populations for the measurement of premorbid IQ (25, 26) and suitable for evaluating educational quality where education is not homogeneous (27).

Previous studies in Peru have shown that older adults with higher reading level scores performed better in executive functions and memory tasks (28). This study concludes that higher literacy levels might reflect a higher quality education received early in life.

These early-life educational experiences (exposure to quality content), also promote the implementation of mechanisms for regulating cognition, such as executive control, which are associated with cognitive reserve and brain resilience mechanisms in later-life (29). Consequently, it seems that limited years of education is not the only risk factor for neurodegenerative diseases, but also low literacy level. Thus, our objective is to analyze the association of literacy level with executive control factors in normal aging. In addition, we analyze the effect of processing speed, years of education and age on executive control. We hypothesize that higher scores in the Word Accentuation Test will predict better performance in executive control tasks, which will be independent of age, years of education, and processing speed. These results will permit us to identify the influence of educational quality on the dynamics of cognition in older adults.

## Methods

### Participants

The initial sample consisted of 121 older adults from public and private senior citizen clubs in the city of Arequipa, Peru. The participants were selected according to the following criteria: not having a history of neurological or psychiatric disease and not having major visual or auditory problems. Besides we excluded participants with Mini-Mental State Examination scores below 27 points if they had more than 7 years of schooling, below 23 points for those with 4–7 years of schooling and below 21 points for those with 1–3 years of schooling; following the used by Custodio & Lira (30). Finally, we excluded participants with scores above 6 points on the Yesavage Geriatric Depression Scale (31) and above 2 points on Reisberg's Global Dementia Staging (32). The final sample was made up of 93 healthy older adults (see Table 1).

**Table 1:** Sociodemographic, clinical and cognitive characteristics of the final sample (*N* = 93), according to the WAT score.

	**WAT scores**		
	**≤24 points *N* = 52**	**>24 points *N* = 41**	***P* value**
Age, mean (SD)	73.42 (8.20)	70 (7.38)	0.064
Education (years), mean (SD)	10.41 (3.84)	13.82 (3.45)	0.000^*^
Sex			
Male, n (%)	6 (5.45)	12 (7.13)	0.422^∧^
Female, n (%)	31 (47.69%)	34 (52.03%)	
MMSE, M (DS)	25.20 (4.43)	28.10 (1.52)	0.001^*^
Geriatric Depression Scale M (SD)	5.39 (2.95)	5.13 (2.93)	0.075
Global Dementia Staging M (DS)	1.43 (0.48).	1.17 (0.47)	0.091
Executive control			
Working memory M (DS)	−0.40 (0.94)	0.41 (0.84)	<0.000^**^
Cognitive Flexibility M (DS)	−0.52 (0.73)	0.47 (0.91)	<0.000^**^
Inhibitory Control M (DS)	−0.51 (0.89)	0.33 (0.92)	<0.000^**^

*WAT, word accentuation test; MMSE, Mini Mental State Examination; M, Media; SD, Standard Deviation*.

* *p < 0.05*.

** *p < 0.001*.

∧ *Chi2*.

*t student.

### Instruments

We used a paper and pencil neuropsychological battery to test various components of executive control. Working memory was assessed with the raw scores of Forward and Backward Digits, as well as of Numbers and Letters from the III Wechsler Scale (33). For cognitive flexibility, we used the raw scores (time) of Trail Making Test B (TMT-B) (34) and the total number of words with “P” (phonological fluency) and animals (semantic fluency) named in 60 s (35). Inhibitory control was evaluated with the Word-Color subtest and interference scores of Stroop Test (36) and the Go/No-Go subtest of the Frontal Assessment Battery (FAB) (37).

Likewise, we evaluated processing speed as a basic attention mechanism that modulates cognition in aging (11). For that purpose, we used the raw scores of Trail Making Test A (time) (TMT-A) (38) and Word and Color subtests of the Stroop Test (36), accordingly the proposal of Perea et al. (39), along with the raw scores of the Digit Symbol subtest from the Wechsler scale (WAIS IV).

Literacy level was measured through the Word Accentuation Test (WAT) (24). We used the Ecuadorian version (26), which showed good internal consistency as well as good test-retest reliability. This test requires the subject to read aloud some low-frequency words presented visually, written in capital letters without an accent mark. The subjects were asked to read the word correctly and mark the tonic accent. We used the 30-item version.

### Procedure

Two sessions of approximately 50 min each were carried out over a period of 2–3 weeks. The assessments were carried out in 2 phases. In the first one, a clinical screening protocol was applied to all subjects, excluding those who did not meet the inclusion criteria. In the second phase, the subjects were administered the neuropsychological battery.

### Statistical Analysis

We performed a Principal Component Analysis (PCA) to reduce the neuropsychological factors and identify executive control and processing speed components. To obtain PCA components, we transformed Direct scores of individual tests into Z scores for standardized data. The number of principal components (PCs) extracted was prespecified using the standard eigenvalues >1 criterion. Top contributors of each rotated PC were defined as those with a factor loading > 0.5.

We used the median obtained in the word accentuation test (Me = 24) to split the participants into two groups (lower score ≤ 24 and higher score > 24) according with Manly et al. (6). Afterwards, Pearson bivariate correlation tests were performed between the PCs found with the WAT score. Finally, we performed a linear regression analysis, in which the PCs were analyzed as dependent variables and the age factor, years of education, WAT scores and composite index of the processing speed factor were the independent variables. The processing was performed using the statistical software SPSS version 24.

### Ethical Aspects

This study is part of a follow-up research project about the impact of literacy level on cognition in preclinical and clinical phases of dementia and was reviewed and approved by the Ethics Committee of the Research unit of San Pablo Catholic University (Acta N° 012.CEDI.UCSP.2020). All participants gave written informed consent in accordance with the declaration of Helsinki.

## Results

### Demographic, Clinical and PC Information on the Participants

The sociodemographic, clinical and PC data (Supplementary Material) of the sample are presented in Table 1. Participants with WAT scores below and above 24 points do not differ significantly in age and sex, but they do in years of education and MMSE (see Table 1). The PCs obtained were inhibitory control (Word-Color and Interference subtests of Stroop Test), cognitive flexibility (TMT-B, semantic [animal] and phonological [letter P] fluency task) and working memory (Forward and Backward Digits and Numbers and Letters). These components explained 72.9% of the variance. In addition, their scores differ significantly between the groups.

### Associations Between Composite Factors and WAT Score

**Figure 1:**
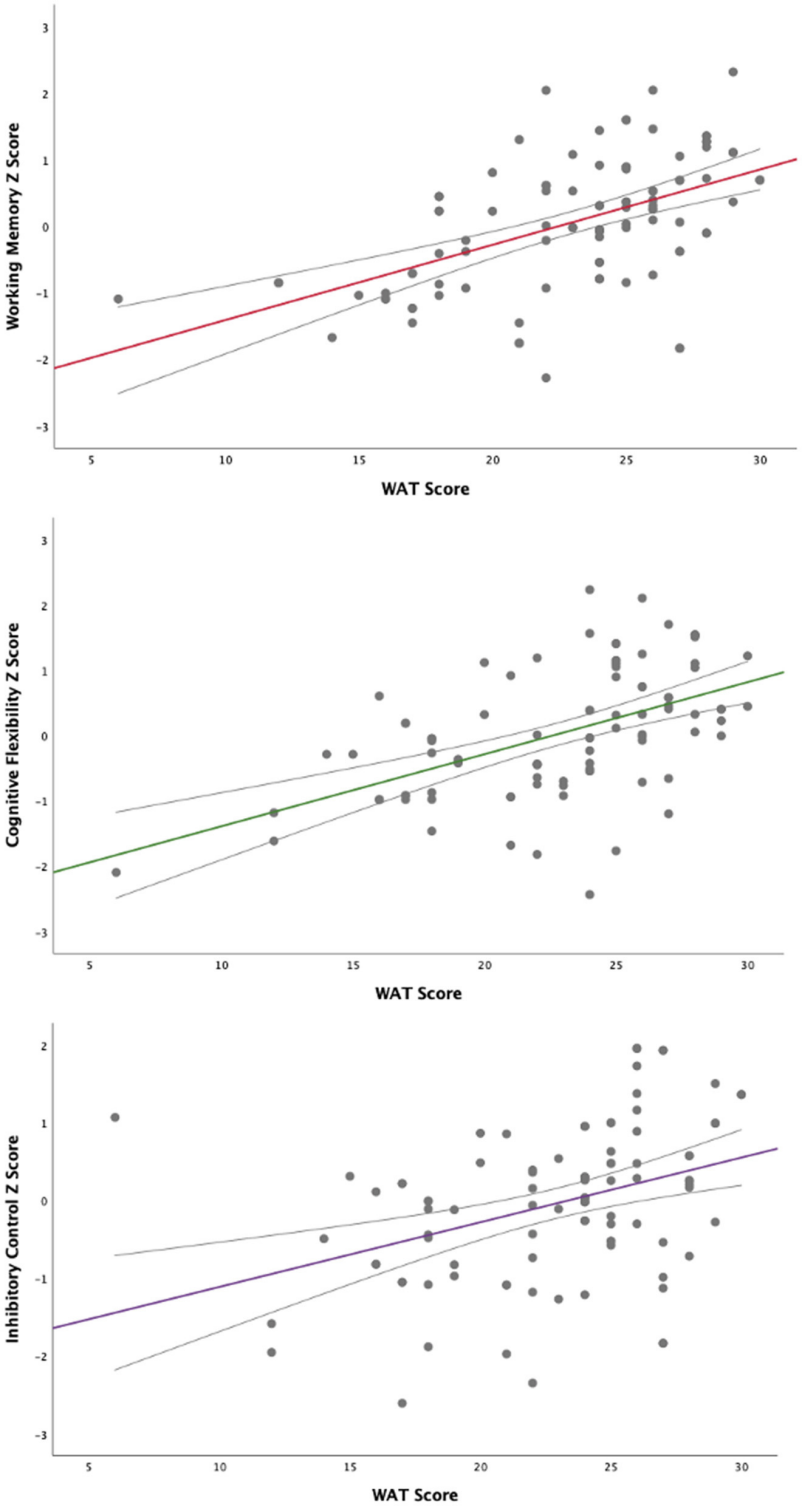
Correlations of executive control PCs with the WAT score.

Pearson bivariate correlation tests showed significant correlations between PC scores of working memory (*r* = 0.527, *p* < 0.000); cognitive flexibility (*r* = 0.521, *p* < 0.000) and inhibitory control (*r* = 382, *p* < 0.000) with the WAT scores across participants (see Figure 1).

### Linear Regression for Principal Components of Executive Control

We performed the linear regression analysis using the composite factor of working memory as the dependent variable and the composite index of processing speed, years of education and age as independent variables. We observed that the WAT and processing speed scores significantly predict the working memory scores and explain 52.7% (adjusted *R*^2^) of the variability in the scores, while years of education and age did not predict working memory scores (see Table 2).

**Table 2:** Linear regression of years of education, age, processing speed and WAT with working memory factor as DV.

							**95% CI for B**	
**Model**	**Predictors**	**USC B**	**SE**	**SC beta**	***t* value**	***p***	**Lower**	**Upper**
**Multivariable**	Education (years)	0.038	0.024	0.154	1.547	0.125	−0.011	0.086
	Age	0.003	0.010	0.025	0.309	0.758	−0.017	0.023
	Processing speed	0.465	0.086	0.469	5.401	<0.001	0.294	0.635
	WAT	0.068	0.017	0.318	3.927	<0.001	0.034	0.103

*WAT, word accentuation test; CI, Confidence Interval; USC B, Unstandarized beta; SE, Standard error; SC, Standardized*.

*R^2^ = 0.527*.

In the case of the cognitive flexibility, we observed that WAT scores, age and processing speed significantly predict the factor scores. In addition, these factors explain 62.3% (adjusted *R*^2^) of the variability (see Table 3).

**Table 3:** Linear regression of years of education, age, processing speed and WAT with cognitive flexibility factor as DV.

							**95% CI for B**	
**Model**	**Predictors**	**USC B**	**SE**	**SC beta**	***t* value**	***p***	**Lower**	**Upper**
**Multivariable**	Education (years)	0.029	0.022	0.121	1.314	0.192	−0.015	0.073
	Age	−0.035	0.010	−0.283	−3.695	<0.001	−0.054	−0.016
	Processing speed	0.357	0.080	0.360	4.461	<0.001	0.198	0.515
	WAT	0.070	0.016	0.330	4.349	<0.001	0.038	0.102

*WAT, word accentuation test; CI, Confidence Interval; USC B, Unstandardized beta; SE, Standard error; SC, Standardized*.

*R^2^ = 0.623*.

Finally, we observed that processing speed and WAT scores predict the inhibitory control factor scores. Similarly, these factors explain 26% (adjusted *R*^2^) of the variations in the factor (see Table 4).

**Table 4:** Linear regression of years of education, age, processing speed and WAT with inhibitory control factor as DV.

							**95% CI for B**	
**Model**	**Predictors**	**USC B**	**SE**	**SC beta**	***t* value**	***p***	**Lower**	**Upper**
	Education (years)	−0.002	0.032	−0.009	−0.069	0.945	−0.066	0.062
**Multivariable**	Age	−0.024	0.014	−0.191	−1.770	0.080	−0.052	0.003
	Processing speed	0.233	0.116	0.229	2.014	0.047	0.003	0.463
	WAT	0.065	0.023	0.301	2.826	0.006	0.019	0.112

*WAT, word accentuation test; CI, Confidence Interval; USC B, Unstandardized beta; SE, Standard error; SC, Standardized*.

*R^2^ = 0.260*.

## Discussion

This study examined the association of literacy level with executive control factors in normal aging by using the word accentuation test (WAT). Our results showed better scores in working memory, cognitive flexibility and inhibitory control in subjects who performed better on the WAT. In addition, we found that scores on this measure, together with the processing speed, better predict executive control scores. These results confirm our hypothesis that literacy level is a better predictor of executive control than years of education or age.

### Predictors of Scores in Executive Control

Our results show that subjects with better WAT scores exhibit a greater capacity to retain and manipulate information (working memory). These results have been confirmed in other studies (40, 41), where working memory, modulated by education, is associated with cognition during aging. This suggests that perhaps formal education strengthens the ability to select, update, and manipulate information available in the working memory, through the promotion of progressively more complex activities in classroom settings, autonomous learning and improvement curricula.

In addition, we found that WAT scores predict performance on inhibitory control measures. Thus, people with higher WAT scores and faster processing speed show a greater ability to inhibit powerful and/or automatic stimuli. Similar results have been found by de Bruin et al. (42) and Hull et al. (43). Their studies showed that inhibitory control is modulated by processing speed and the type of task used for its assessment. Some tasks are more demanding than others and therefore require more inhibitory control mechanisms to be carried out correctly.

Likewise, we found higher scores for the cognitive flexibility factor when the WAT score is higher, and performance is better predicted by this, together with age and processing speed, as has been reported in research by Gallen et al. (12). We observed that participants who are younger and those with higher literacy (higher WAT scores) have a greater capacity to change attentional and mental sets (44). This is a fundamental aspect in accomplishing complex cognitive tasks (45).

These results could help us to understand the importance of executive control mechanisms in brain and cognitive aging, showing a bigger impact in the case of low-quality education. As a result, we would observe a weakening in mechanisms associated with brain resilience, like white matter integrity and cortical thinning, which could contribute to cognitive decline in the following years (46) and cognitive reserve (47) used to compensate for cognitive changes with an alternative network or better processing strategy. Therefore, people with low literacy levels could perform worse on tasks and have a greater risk of cognitive impairment and dementia.

### Literacy Level, Executive Control and Education

Literacy level allows us to explain the scores on the executive control tasks. Nonetheless, it should be noted that processing speed is a mechanism with a significant effect on adult cognitive performance (11, 48). Our data show that the measurement and analysis of literacy level (educational quality) and processing speed can more clearly explain cognitive changes in older adults than years of education. That is, exposure to quality content or being exposed to a more enriching education might make the execution of general processing tasks and those that involve executive control more efficient.

If individuals with a more efficient processing capacity faces a neurodegenerative event, they can generate brain and cognitive compensatory strategies that minimize the impact of the pathology (49–52) and reduce the risk of cognitive impairment or dementia. This could help to achieve satisfactory aging, through the implementation of cognitive reserve mechanisms (53), and thus protect against neurodegenerative pathology. This is an aspect we might not see in subjects with low literacy levels.

This first look at studying the literacy level in Peru can help us to explain the cognitive and behavioral variants observed in older adults who have different scores and performances despite having the same years of education. We consider that literacy level is a more accurate measure than years of education when assessing the effect of formal education on cognition and, consequently, as a protective factor for the development of neurodegenerative pathologies. This is particularly important in settings such as Peru where the quality of education is highly variable, educational quality statistics are below international standards (54) and where the education received depends on socioeconomic status and area of residence (55).

### Limitations

Our findings are interesting, but they are not without limitations. As we do not have a group of subjects with a clinical diagnosis (mild cognitive impairment or mild Alzheimer's), we cannot establish a point of comparison with impairment at the executive and cognitive level derived from the WAT score. Furthermore, since it is a cross-sectional study, we cannot precisely establish the protective factor of literacy level. We need to carry out longitudinal studies to estimate the level of protection participants have against deterioration. Furthermore, the Socio-Economic Status (SES) and Intelligence Quotient (IQ) have not been considered in the present study and could be associated with the results obtained (56). The impact of SES on cognition could be mitigated by years of schooling, literacy level, and other environmental factors (57), so they need to be evaluated in future research. Finally, as we do not have a standardized WAT test in Peru, we used the Ecuadorian version. Despite similarities between Andean nations in many aspects (e.g., culturally, educational, etc.), it is necessary to validate the WAT in the Peruvian context.

### Conclusions

We can conclude that literacy level, measured through the WAT, is significantly correlated with executive control processes in elderly people in Peru. One interpretation of this is that higher educational quality in young age (as indicated by literacy scores in older age) favor a better development of executive control over the life span.

In this context, we consider that in Peru it is not only illiterate people who have a higher risk of developing dementia, but also adults with lower scores in literacy level and quality of education measures. Consequently, there is a need to develop programs aimed at improving educational quality from the early years of education. This is in addition to the implementation of cognitive intervention programs that favor cognitive mechanisms such as attention and executive function among our adults.

## Data Availability Statement

The raw data supporting the conclusions of this article will be made available by the authors, without undue reservation.

## Ethics Statement

The studies involving human participants were reviewed and approved by the Ethics Committee of the Research unit of San Pablo Catholic University (Acta N° 012.CEDI.UCSP.2020). Written informed consent to participate in this study was provided by the patients/participants.

## Author Contributions

MS-A: designed the study, performed the statistical analysis and the preparation, and final approval of the manuscript. NL: participated in the statistical analysis and in the preparation and final revision of the manuscript. CR-F and VB-H: analyzed the data and participate in the preparation and final revision of the manuscript. SF-G: designed the study and revised and approved the manuscript. All authors contributed to the article and approved the submitted version.

## Conflict of Interest

The authors declare that the research was conducted in the absence of any commercial or financial relationships that could be construed as a potential conflict of interest.

## Publisher's Note

All claims expressed in this article are solely those of the authors and do not necessarily represent those of their affiliated organizations, or those of the publisher, the editors and the reviewers. Any product that may be evaluated in this article, or claim that may be made by its manufacturer, is not guaranteed or endorsed by the publisher.
