# Supplementary material for: Literacy Level and Executive Control in Healthy Older Peruvian Adults
Source: Front Neurol. 2021 Aug 26;12:629048. doi: 10.3389/fneur.2021.629048 (PMC8426511; doi:10.3389/fneur.2021.629048)
Supplement: Supplementary file 1 [file Table_1.DOCX]

Supplementary Material

**Principal components extracted: executive functions and processing speed factors**

**Executive functions factors**

The KMO index was .785 and Bartlett’s sphericity (χ^2^=373.024, p<.000) indicated that data were suitable for factor analysis. The principal component analysis showed a solution of 3 factors: cognitive flexibility, inhibitory control, and working memory (Table 1). There were factorial weights ranging from .506 to .955 and an explained variance of 72%.

**Processing speed factor**

The KMO index was .701 and Bartlett’s sphericity (χ^2^=110.148, p<.000) indicated that data were suitable for factor analysis. The principal component analysis showed an unifactorial solution (Table 1) with factorial weights ranging from .829 to .891 and an explained variance of 74%.

**Table 1.** Component’s loadings in extracted factors

| **Components**  **extracted**  **Neuropsychological**  **test** | Processing Speed | EF  Cognitive Flexibility | EF  Inhibitory  control | EF  Working Memory |
| --- | --- | --- | --- | --- |
| Stroop word | .891 |  |  |  |
| Stroop Color | .829 |  |  |  |
| TMT A time | -.852 |  |  |  |
| Fluency P |  | .655 |  |  |
| Fluency Animals |  | .793 |  |  |
| TMT B time |  | -.813 |  |  |
| Go/No Go |  | .671 |  |  |
| Stroop W-C |  |  | .738 |  |
| Stroop interference |  |  | .938 |  |
| Digit Forward |  |  |  | .866 |
| Digit backward |  |  |  | .876 |
| Numbers and Letters |  |  |  | .506 |

Abbreviations: EF, Executive function; TMT B, Trail Making Test B; Stroop W-C, Stroop word color
